# 

**Published:** 1891-12-19

**Authors:** 


					'homas s Hospital
1RWICH HOSPI TAL
Clascoiy Western Infirmary
WITH EXTRA NURSING SUPPLEMENT.
For IKFLUElfZA,?BOTBIL for the last
two years has established itself throughout
Earope and Amerioa as the most perfeot antidote,
the most certain preventive
, ? I, and approved cure of the
t ?'a8es' BOVEIL (th? produot of prime ox
rWiotrt ?6 system that stamina and vitality whioh can
" HuQjpf,1*? influenoe of the seourge.and carries the feeblest
Ka the climax of the attack t j a rapid recovery.
"? ?? ??-D.il, Post."?Sir,?Oa
way^Hit weennfea? SSV Teacups, full of BOVSII, mid-
cure any one ?!& I St at mght' 1 baliev0 this will
The Grange. Rock P^. (S?ned) Qa:a%fc
TljW
T
c
No. 273. Vol. XI.] ^Saturday,*
December 19th, 1891.
[One Penirr.

				

